# Blood Pressure Control and Associations with Social Support among Hypertensive Outpatients in a Developing Country

**DOI:** 10.1155/2021/7420985

**Published:** 2021-04-03

**Authors:** Luu Quang Thuy, Nguyen Hoang Thanh, Le Hong Trung, Pham Huy Tan, Hoang Thi Phuong Nam, Pham Thi Diep, Tran Thi Ha An, Bui Van San, Tran Nguyen Ngoc, Ngo Van Toan

**Affiliations:** ^1^Vietnam Viet-Duc Hospital, Hanoi 100000, Vietnam; ^2^Hanoi Medical University, Hanoi 100000, Vietnam; ^3^Vinh Phuc General Hospital, Vinh Phuc 100000, Vietnam; ^4^Hanoi Medical University Hospital, Hanoi 100000, Vietnam; ^5^National Geriatric Hospital, Hanoi 100000, Vietnam; ^6^Faculty of Health Sciences, Thang Long University, Hanoi 100000, Vietnam; ^7^National Institute of Mental Health, Bach Mai Hospital, Hanoi 100000, Vietnam

## Abstract

Relationships between social support characteristics with blood pressure control and recommended behaviors in Vietnamese hypertensive patients have not been investigated. This study is aimed at examining the role of social support characteristics in hypertension control and behaviors. Patients with hypertension (*n* = 220) in Hanoi, Vietnam, were recruited into a cross-sectional study. Both functional and structural characteristics of social support and network were examined. Results showed that increasing total network size was related to 52% higher odds of uncontrolled hypertension (adjusted OR = 1.52, 95%CI = 1.22 − 1.89). Higher network sizes on the provision of information support related to advice, emotional support related to decisions, and practical support related to sickness were associated with lower odds of uncontrolled hypertension. Every additional 1% of the percentage of network members having hypertension decreased 2% the odds of uncontrolled hypertension (adjusted OR = 0.98, 95%CI = 0.96 − 1.00). A 1% additional network members who were living in the same household was associated with a decrease of 0.08 point of behavioral adherence score (coef. = −0.08; 95%CI = −0.12 − 0.03). Meanwhile, a 1% increase of network members who were friends on the provision of practical support related to sickness and jobs was related to an increase of 0.10 point and 0.19 point of behavioral adherence score (coef. = 0.10; 95%CI = 0.04 − 0.17 and coef. = 0.19; 95%CI = 0.06 − 0.32, respectively). The current study suggested that further interventions to improve hypertension management should address the potential effects of social network characteristics.

## 1. Introduction

People are now living longer but more vulnerable to noncommunicable diseases (NCDs) [1]. Hypertension is one of the most common NCDs, with more than one billion people experiencing this disease worldwide [2, 3]. Timely diagnosis and appropriate treatment are essential to control hypertension as well as prevent its severe complications such as cardiovascular diseases, stroke, or kidney failure [4]. However, it is estimated that more than half of patients cannot control their blood pressure, even in hospitals where hypertensive medications and follow-up care are provided freely [5–7]. Nonmedication adherence and unhealthy lifestyles are major reasons of uncontrolled hypertension [8], suggesting the matter of the fact that healthcare providers have a limited role in managing this disease [9].

Changing healthy behaviors seems not to be easy because it requires enormous efforts and motivations from each individual [10]. Motivations can be improved when patients receive support and encouragement from their community or family [11, 12]. In other words, patients' social context is critically important for their health enhancement. The social network, or social relationships, is defined as “a social structure made of individuals (or organizations) that represents “nodes”, and they are associated with one or more types of interdependency, such as friendship, common interests, work, knowledge, prestige, and many other interests [13]. Network members of one individual can be anyone who has interaction with this individual (partner, siblings, parents, friends, etc.). Previous literature underlined the association between social support/network and reduced risk of physical and mental health problems as well as mortality [14–17]. People with well-social connectedness were more likely to have healthy behaviors and better life satisfaction [18, 19]. These relationships have been observed in many patients groups with different health conditions such as overweight/obesity, substance use, depression, and cancer screening [20–24]. Owning a larger and more diverse social network is found to improve self-esteem, which can alleviate the physiological reactivity, improve immune system, and buffer psychological distress [25–27]. In low- and middle-income countries, prior studies suggested the critical role of social support as safety nets, particularly for middle-age and older adults [28, 29].

In the hypertensive community, several studies indicated the positive influence of both functional (e.g., social, emotional, or practical support) and structural (e.g., network size or type of relationship) social support and network characteristics of patients on the prevalence of hypertension, blood pressure control, or hypertensive complications. For example, a study in Korean older adults revealed that people with a larger social network size were less likely to suffer from hypertension [30], while another study in Nigeria showed that a high degree of family support was associated with better hypertension control [31]. Moreover, social interventions have been proposed to be more useful to prevent hypertension than interventions that only concentrated on individual factors [32]. Therefore, exploring the functional and structural characteristics of social support in patients with high blood pressure is vital for appropriate hypertension management.

In this study, we aimed to examine the role of social support and network characteristics in hypertension control and adherence of recommended behaviors among hypertensive patients managed at an urban hospital in Hanoi, Vietnam.

## 2. Materials and Methods

### 2.1. Study Settings, Participants, and Sampling Procedure

Data of this analysis were from a cross-sectional study implemented at an urban hospital in Hanoi, Vietnam, from August to October 2019. There were approximately two thousand outpatients with hypertension who registered for hypertension management in this hospital. The protocol of this study was approved by the institutional review board of the hospital. We recruited outpatients aged 18 years or above who were confirmedly diagnosed with high blood pressure based on the guideline of the Vietnam Ministry of Health (persistently systolic blood pressure level of ≥140 mmHg and/or persistently diastolic blood pressure level ≥ 90 mmHg) [33]. Other eligible criteria included (1) visiting outpatient clinics of the hospital during the study period, (2) accepting to be participants of this study and giving their written informed consent. Patients were excluded if they were inpatients or had disabilities and other cognitive impairments which affected their ability to answer the face-to-face interview.

To calculate the sample size, we employed the formula to estimate a proportion with specified relative precision with confidence level = 95% and expected proportion of uncontrolled hypertension among treated hypertensive patients = 64.0% (according to a previous study in Central Vietnam [34]). The total sample size was 217 patients. In practice, we recruited 225 patients to prevent those who did not complete the interview. Data of 220 people were included in the data analysis (completion rate = 97.8%).

A convenient sampling procedure was performed to recruit patients. First, patients who visited outpatient clinics during the study period for regular hypertension examination and treatment were screened the eligible criteria (such as physical and mental health) by the physicians at the clinics. Then, when they completed all examination procedures (including blood pressure measures), patients were invited to become study participants by the research team and go to a private counseling room for protecting their confidentiality. They were provided a short description of the study and their rights as participants. A face-to-face interview was performed afterward, following by weight and height measures.

### 2.2. Data Collection and Measurement

The data collection team included undergraduate medical students from the Hanoi Medical University. Before collecting the data, they participated in intensive training sessions about interview skills with patients having high blood pressure, as well as the data collection procedure to ensure their consistency in data collection. They also participated in the pilot study with five patients to assure that they followed correctly the data collection guideline. A structured questionnaire was developed and piloted in these five patients to check the validity and reliability of the questionnaire. During the data collection, one student was in charge of the interview, and another student measured the weight and height of patients. Following variables were collected:

#### 2.2.1. Uncontrolled Hypertension

When patients had regular medical examinations, their office blood pressure was measured by the hospital's physicians using the Japanese Alpk2 sphygmomanometer. After completing the interview, our data collector extracted this information from the patient's medical record. Patients having a systolic blood pressure level of ≥140 mmHg and/or diastolic blood pressure level ≥ 90 mmHg were classified into the “uncontrolled” group; otherwise, they were categorized into the “controlled” group [33].

#### 2.2.2. Behavioral Adherence

Eight items from the Condition-specific Recommendations and Adherence scale were used to assess the adherence of patients with different recommended behaviors for patients with hypertension [35]. These behaviors consisted of (1) take prescribed medication daily, (2) follow a low-salt diet, (3) follow a low-fat diet, (4) exercise regularly, (5) stop/cut down on smoking, (6) cut down on alcohol, (7) cut down on stress, and (8) carry supplies needed for self-care. There were six response options for each behavior: 0 = none of the time, 1 = a little of the time, 2 = some of the time, 3 = a good bit of the time, 4 = most of the time, and 5 = all of the time. The total score was 0-40, and patients with 32/40 points were categorized as “overall health behavior adherence”; otherwise, they were categorized into the “overall non-adherence” group [35].

#### 2.2.3. Social Support and Network Characteristics

In this study, we examined both functional and structural network characteristics of hypertensive patients by using a name generator method, which is one of the most common approaches to explore the individual's egocentric network [36, 37]. An egocentric network is a network that considers each patient (i.e., “ego”) a center of the network. A person having relationships with the ego was recorded as a network member (i.e., alter) [36, 37]. This method helps to figure out the social network of each participant as well as identify the general characteristics of each network member [36, 37]. In this study, we adapted the previous study [38] and used five questions, including

1. “In the last six months, who was the one that advises you when you had problems?” (informational support related to advice)
2. “In the last six months, who was the one that could offer their help when you were sick?” (practical support related to sickness)
3. “In the last six months, who was the one that could offer their emotional support when you were feeling unwell?” (emotional support related to discomfort)
4. “In the last six months, who was the one that could help you to perform the housework?” (practical support related to jobs)
5. “In the last six months, who was the one that could discuss important matters with you?” (emotional support related to decisions)

Patients were asked to call as many names as they could remember for each question. The name could be duplicated between questions but could not be duplicated within each question. We then calculate the participants' network size by summing the total number of unique alters in the questionnaire. We also asked patients to report some general characteristics of each network member, including gender, relationship with the participant (e.g., family member, people living in the same household, and friend or others), living place, contact frequency (daily/weekly/monthly/yearly), and whether having hypertension or not. Marital status and whether participating in social activities or not were also examined.

#### 2.2.4. Other Covariates

Other information was collected including age, gender, education, occupation, living area, smoking and alcohol drinking status, comorbidities, weight, height, body mass index, and health-related quality of life. The Short-form 12 version 2 (SF-12v2) instrument investigates HRQoL of hypertensive patients regarding physical component (PCS-12) and mental component (MCS-12) [39, 40].

### 2.3. Statistical Analysis

We used the Stata software 14.0 for data analysis. Descriptive analyses were conducted to examine the differences in social network characteristics between patients with controlled/uncontrolled hypertension and between patients with behavioral adherence/nonadherence. Chi-squared tests and Mann–Whitney tests were performed as appropriate. Multivariate logistic and tobit regression models were used to explore the association of network variables with uncontrolled hypertension (yes/no) and behavioral adherence score. In this study, we conducted four models for each dependent variable. Model 1 was adjusted for age and sex, while model 2 was adjusted for age, sex, and quality of life (PCS-12 and MCS-12). Model 3 was additionally adjusted for body mass index and number of comorbidities, while model 4 was additionally adjusted for smoking and alcohol use. Multicollinearity was checked by using variance inflation factor (VIF). VIF results showed no multicollinearity among variables. Akaike information criterion (AIC) and Bayesian information criterion (BIC) were then calculated to examine the sensitivity of the models. The level of statistical significance was set at the 5% level.

## 3. Results

There were 220 hypertensive patients included in the study. The mean age was 64 years (SD 8.9 years). There were 10.9% of patients having lower secondary education level, 44.5% being current self-employed, and 40.5% being retired. Most participants (81.8%) were living with their partners and living in urban areas (85.5%). There were 54.6% reporting that participated in social activities. The majority of patients did not smoke (60.9%). The rate of uncontrolled hypertension was 40.5%, while the rate of adherence with recommended behaviors was 30.4%.

Table 1 shows the social support and network characteristics of hypertensive patients. Patients with uncontrolled hypertension had a significantly higher percentage of network members living in the same households who offered information support (*p* < 0.05) compared to those with controlled hypertension. Meanwhile, participants having adherence with recommended behaviors had a significantly higher percentage of network members who are friends on the provision of practical support-related jobs than that of their nonadherence counterparts (*p* < 0.05). Moreover, in the behavioral adherence group, the percentage of patients participating in the social activities (66.7%) was significantly higher than that of the behavioral nonadherence group (50.3%, *p* < 0.05).

Table 2 reveals that overall, increasing total network size was related to 52% higher odds of uncontrolled hypertension (adjusted OR = 1.52, 95%CI = 1.22 − 1.89). However, higher network sizes on the provision of information support related to advice, emotional support related to decisions, and practical support related to sickness were associated with lower odds of uncontrolled hypertension. In model 4, every additional 1% of the percentage of network members who were daily contact on the provision of practical support related to sickness was related to 2% higher odds of uncontrolled hypertension (adjusted OR = 1.02, 95%CI = 1.00 − 1.05), while every additional 1% of the percentage of network member having hypertension decreased 2% the odds of uncontrolled hypertension (adjusted OR = 0.98, 95%CI = 0.96 − 1.00).

Table 3 depicts that regarding networks on informational support related to advise, an additional 1% in the percentage of network members who were living in the same household was associated with a decrease of 0.08 point of behavioral adherence score (coef. = −0.08; 95%CI = −0.12 − 0.03). Meanwhile, an 1% increase of network members who were friends on the provision of practical support related to sickness and jobs was related to a 0.10 point and 0.19 point of behavioral adherence score (coef. = 0.10; 95%CI = 0.04 − 0.17 and coef. = 0.19; 95%CI = 0.06 − 0.32, respectively). Higher social network sizes on emotional support related to decisions and practical support related to sickness were associated with higher behavioral adherence score, but in model 4, these associations were not statistically significant (*p* > 0.05). Notably, patients participating in social activities had significantly higher behavioral adherence score compared to those not participating in such activities (coef. = 2.56; 95%CI = 0.99 − 4.13).

## 4. Discussion

Our study informs an insight into the relationship between functional and structural characteristics of social support and uncontrolled hypertension as well as adherence with recommended behaviors among hypertensive patients in the urban setting of Vietnam. The findings of this study indicated that both functional and structural network characteristics were associated with hypertension control and behavioral adherence. For example, higher network size on the provision of informational support, emotional support related to decisions, and practical support related to sickness was independently associated with a lower likelihood of uncontrolled hypertension. Moreover, social activity participation appeared to be independently related to a higher behavioral adherence score.

In this study, we have observed that an overall large network size would increase the risk of uncontrolled blood pressure. However, our result differed from a prior study in Korean and American older adults, which showed that hypertensive patients having a large network size would have lower odds of uncontrolled hypertension [9, 30]. It might justify this difference in sample characteristics and culture. Indeed, 30% of our patients were still employed (<60 years old), while the patients in other studies were aged 60 years or more. Nonetheless, we found that lower degrees of information support, emotional support, and practical support was considerably related to uncontrolled hypertension, underlining the critical role of different social network aspects in managing hypertension, that echoes previous studies that indicated that insufficient social support was correlated with a higher risk for cardiovascular diseases [38, 41]. Cornwell et al. found that emotional support was associated with a lower risk of uncontrolled hypertension [9]. These authors also revealed that patients who frequently discussed health problems with their network were less likely to have uncontrolled hypertension [9]. However, our study has contradicted this finding since increasing network members who could daily talk about sickness would increase the risk of uncontrolled hypertension. We assumed that regular discussion or communication about health problems might cause the relationships more tense or conflicted, resulting in more stress for patients [42]. On the other hand, patients receiving practical support to their housework/jobs from networks with higher percentages of hypertensive members were less likely to suffer uncontrolled hypertension. This result might emphasize the fact that having peers with hypertension who helped patients in daily activities would be more beneficial to control the disease rather than having a more extensive network size but increasing stress levels in their lives.

Regarding behavioral adherence, we found that patients having more practical support from friends were more likely to adhere to behavioral recommendations. Moreover, higher percentages of network members living in the same household would decrease adherence. In other words, the finding revealed that patients with a centralized network to household members were at higher risk of nonadherence. Another notable finding is that participating in social activities would increase the level of behavioral adherence. It should be noted that two-thirds of our samples were 60 years old or more. Previous literature suggested that older people should be encouraged to have an extensive social network and participate in social activities to promote their autonomy and social roles, which could increase their physical and mental health [43–45]. These findings suggest the critical social network-based preventive strategies to encourage behavioral adherence among hypertensive patients. More studies should be elucidated to clarify these relationships.

The findings of this study pose several implications for public health and clinical practitioners. First, physicians should pay attention to the social relationships of hypertensive patients and the influences of these relationships with hypertensive treatment and behaviors. Awareness of patients' social support and social network can help to develop effective and tailored interventions based on the network characteristics for improving treatment outcomes and lifestyle behaviors. Second, our results support the development of tailored social network-based interventions to prevent uncontrolled blood pressure among hypertensive patients. Second, further studies should be warranted to examine the role of different network characteristics in the treatment progress and the development of hypertensive complications. Third, we believed that social support and network characteristics also had an important role in the onset of hypertension [9, 46], which should be resolved in future interventions to prevent hypertension in the community. Moreover, further behavioral change interventions should take into account the social network, which has been shown in the previous literature that can help to improve the adoption of public health interventions [47].

This study has strengths, including the use of several validated instruments such as SF-12v2 and Condition-specific Recommendations and Adherence scale, which allowed study findings to be comparable to previous research in the world. Moreover, we employed a name generator, which is among the most common method to explore the egocentric network of each person [36, 37]. The associations between social network variables and our outcomes of interests were also adjusted for well-known potential confounders. However, several methodological limitations should be acknowledged. First, the nature of the cross-sectional design in this study did not allow us to have a causal conclusion regarding the associations. Second, patients should recall the names in their social network, which might lead to recall bias. Third, we performed this study in only one hospital, which might decrease our ability to generalize the finding to the general hypertensive population in Vietnam.

## 5. Conclusions

The current study showed that higher network sizes on the provision of informational support, emotional support related to decisions, and practical support related to sickness were associated with a lower likelihood of uncontrolled hypertension. Findings also revealed that social support and network characteristics were related to hypertension control and adherence with recommended behaviors. Further interventions to improve hypertension management should address the potential effects of social network characteristics.

## Figures and Tables

**Table 1:** Social support and network characteristics of hypertensive patients according to control of hypertension conditions and behavioral adherence conditions.

Characteristics of social network	Controlled hypertension^a^			Behavioral adherence^a^		
	Yes (*n* = 131)	No (*n* = 89)	*p* value	Yes (*n* = 66)	No (*n* = 151)	*p* value
Total network size	5.3 ± 6.5	4.8 ± 7.8	0.92	5.8 ± 7.1	5.5 ± 6.4	0.70
Providing informational support related to advice						
Network size	2.1 ± 2.1	4 ± 6.5	0.33	5.4 ± 4.6	2.2 ± 1.9	0.59
Percentage of network members who are daily contact	21.1 ± 25.8	39.4 ± 42.3	0.39	42 ± 36.4	25.2 ± 17.6	0.23
Percentage of network members who are family members	17.1 ± 23.3	34.9 ± 40	0.25	38.5 ± 34.2	21.3 ± 15.7	0.34
Percentage of network members who are friends	13.6 ± 9.8	32.1 ± 27.6	0.35	31.1 ± 29.1	12.8 ± 11	0.65
Percentage of network members who are living in the same household	6.3 ± 15.6	21.7 ± 32.7	0.03	30.7 ± 15.2	12.8 ± 4.2	0.17
Percentage of network members who are having hypertension	7.1 ± 6.1	21 ± 21.5	0.32	22.8 ± 16	6.8 ± 5.3	0.82
Providing emotional support related to discomfort						
Network size	2.9 ± 3.5	2.8 ± 4.2	0.31	3.3 ± 5.4	2.6 ± 2.8	0.60
Percentage of network members who are daily contact	73.6 ± 43.7	72 ± 44.6	0.78	73 ± 44.4	73 ± 43.9	0.90
Percentage of network members who are family members	57.9 ± 47.8	64.5 ± 47.2	0.32	61.3 ± 47.8	60.2 ± 47.6	0.88
Percentage of network members who are friends	18 ± 36.6	9.9 ± 28.9	0.07	9.9 ± 27.9	17.1 ± 36.2	0.18
Percentage of network members who are living in the same household	43.9 ± 45.4	52.5 ± 46.6	0.23	45.3 ± 45	48.6 ± 46.7	0.74
Percentage of network members who are having hypertension	14.1 ± 29.4	12.7 ± 29	0.50	19.8 ± 35.5	10.8 ± 25.8	0.08
Providing emotional support related to decisions						
Network size	2.7 ± 3.3	2.8 ± 4.9	0.45	3.3 ± 5.5	2.5 ± 3.1	0.20
Percentage of network members who are daily contact	75.4 ± 41.2	71.2 ± 43	0.51	74.6 ± 39.8	73.4 ± 42.8	0.98
Percentage of network members who are family members	79.8 ± 40.1	75.3 ± 43.4	0.45	80.3 ± 40.1	77.2 ± 41.9	0.59
Percentage of network members who are friends	2.3 ± 15	2.1 ± 13.9	0.97	4.3 ± 20	1.3 ± 11.5	0.15
Percentage of network members who are living in the same household	55.7 ± 44.3	55.8 ± 44.9	0.92	52.4 ± 42	57.6 ± 45.7	0.44
Percentage of network members who are having hypertension	13.2 ± 28.8	14 ± 31.2	0.77	15.6 ± 30.1	12.6 ± 29.8	0.10
Providing practical support related to sickness						
Network size	3.5 ± 3.1	3.6 ± 3.9	0.43	3.8 ± 3	3.4 ± 3.6	0.10
Percentage of network members who are daily contact	78.7 ± 38.1	79.9 ± 37.3	0.87	74.5 ± 39.7	81.2 ± 37	0.16
Percentage of network members who are family members	81.2 ± 36.6	81.6 ± 38	0.75	80.9 ± 36	81.2 ± 38	0.56
Percentage of network members who are friends	3.6 ± 15.5	2.4 ± 13.7	0.37	4.3 ± 14.4	2.7 ± 15	0.09
Percentage of network members who are living in the same household	58.1 ± 41.1	62.8 ± 40.6	0.42	56.5 ± 38.6	62.2 ± 41.8	0.31
Percentage of network members who are having hypertension	10 ± 21.9	9.1 ± 23.3	0.29	10.6 ± 21.2	9.2 ± 23.2	0.14
Providing practical support related to jobs						
Network size	1.8 ± 1.8	2.4 ± 4	0.16	2.4 ± 4.5	1.9 ± 1.9	0.60
Percentage of network members who are daily contact	69.3 ± 45.9	69.6 ± 45.1	0.93	65.9 ± 46.3	71 ± 45.1	0.43
Percentage of network members who are family members	69.6 ± 45.8	71.2 ± 45.1	0.85	68.9 ± 46.2	70.9 ± 45.2	0.82
Percentage of network members who are friends	0.8 ± 8.7	1 ± 9.1	0.79	2.8 ± 16.1	0.0 ± 0.0	0.03
Percentage of network members who are living in the same household	62 ± 47.4	60.5 ± 46.5	0.70	55.7 ± 47.3	64.2 ± 46.7	0.21
Percentage of network members who are having hypertension	14.1 ± 32.1	8.4 ± 23.6	0.20	10.7 ± 25.6	11.6 ± 29.8	0.36
Living with spouse/partners (%)	80.9	83.2	0.67	80.3	82.8	0.66
Participation in social activities (%)	56.5	51.7	0.48	66.7	50.3	0.03

^a^Expressed as mean ± SD.

**Table 2:** Relationship between social network characteristics and controlled blood pressure.

Characteristics of social network	Uncontrolled hypertension (yes/no)^a^			
	OR (95% CI)	OR (95% CI)	OR (95% CI)	OR (95% CI)
	Model 1^b^	Model 2^c^	Model 3^d^	Model 4^e^
Total network size	1.45^∗∗∗^ (1.20-1.77)	1.47^∗∗∗^ (1.21-1.80)	1.47^∗∗∗^ (1.20-1.81)	1.52^∗∗∗^ (1.22-1.89)
Providing informational support related to advice				
Network size	0.77^∗∗∗^ (0.65-0.90)	0.76^∗∗∗^ (0.64-0.90)	0.77^∗∗∗^ (0.65-0.91)	0.76^∗∗∗^ (0.64-0.91)
Percentage of network members who are daily contact	1.00 (0.98-1.02)	1.00 (0.98-1.01)	1.00 (0.98-1.01)	1.00 (0.98-1.01)
Percentage of network members who are family members	1.01 (0.99-1.02)	1.00 (0.99-1.02)	1.00 (0.99-1.02)	1.00 (0.99-1.02)
Percentage of network members who are friends	1.00 (0.99-1.02)	1.00 (0.99-1.02)	1.00 (0.98-1.02)	1.00 (0.98-1.02)
Percentage of network members who are living in the same household	1.02^∗^ (1.00-1.04)	1.02 (1.00-1.04)	1.02 (0.99-1.04)	1.01 (0.99-1.04)
Percentage of network members who are having hypertension	1.00 (0.98-1.02)	1.00 (0.98-1.03)	1.01 (0.98-1.03)	1.01 (0.99-1.04)
Providing emotional support related to discomfort				
Network size	0.92 (0.79-1.06)	0.91 (0.78-1.06)	0.92 (0.78-1.07)	0.89 (0.76-1.05)
Percentage of network members who are daily contact	0.99^∗^ (0.97-1.00)	0.99 (0.97-1.01)	0.99 (0.97-1.01)	0.99 (0.97-1.01)
Percentage of network members who are family members	1.01 (0.99-1.03)	1.01 (0.99-1.03)	1.00 (0.98-1.02)	1.00 (0.98-1.02)
Percentage of network members who are friends	1.00 (0.98-1.01)	0.99 (0.98-1.01)	0.99 (0.98-1.01)	0.99 (0.97-1.01)
Percentage of network members who are living in the same household	1.00 (0.99-1.02)	1.01 (0.99-1.02)	1.01 (0.99-1.02)	1.01 (0.99-1.03)
Percentage of network members who are having hypertension	1.00 (0.98-1.01)	1.00 (0.98-1.01)	0.99 (0.98-1.01)	0.99 (0.98-1.01)
Providing emotional support related to decisions				
Network size	0.86^∗∗^ (0.74-0.99)	0.84^∗∗^ (0.73-0.98)	0.85^∗∗^ (0.73-0.99)	0.86^∗∗^ (0.74-1.00)
Percentage of network members who are daily contact	0.99 (0.98-1.01)	0.99 (0.97-1.01)	0.99 (0.97-1.01)	0.99 (0.97-1.01)
Percentage of network members who are family members	1.00 (0.99-1.02)	1.00 (0.99-1.02)	1.00 (0.99-1.02)	1.00 (0.99-1.02)
Percentage of network members who are friends	0.97 (0.93-1.02)	0.98 (0.93-1.02)	0.97 (0.93-1.02)	0.97 (0.93-1.02)
Percentage of network members who are living in the same household	1.00 (0.99-1.02)	1.00 (0.99-1.02)	1.00 (0.99-1.02)	1.00 (0.99-1.02)
Percentage of network members who are having hypertension	1.02^∗^ (1.00-1.03)	1.01^∗^ (1.00-1.03)	1.02^∗^ (1.00-1.03)	1.02^∗^ (1.00-1.03)
Providing practical support related to sickness				
Network size	0.81^∗^ (0.66-1.00)	0.80^∗∗^ (0.64-1.00)	0.78^∗∗^ (0.62-0.98)	0.75^∗∗^ (0.59-0.96)
Percentage of network members who are daily contact	1.02∗ (1.00-1.04)	1.02^∗^ (1.00-1.04)	1.02^∗∗^ (1.00-1.05)	1.02^∗∗^ (1.00-1.05)
Percentage of network members who are family members	0.98 (0.96-1.00)	0.98 (0.96-1.00)	0.98 (0.96-1.01)	0.98 (0.96-1.01)
Percentage of network members who are friends	0.98 (0.95-1.02)	0.98 (0.95-1.02)	0.98 (0.95-1.02)	0.99 (0.95-1.03)
Percentage of network members who are living in the same household	1.01 (0.99-1.02)	1.01 (0.99-1.02)	1.00 (0.99-1.02)	1.00 (0.98-1.02)
Percentage of network members who are having hypertension	1.00 (0.98-1.02)	1.00 (0.98-1.02)	1.00 (0.98-1.02)	1.00 (0.98-1.03)
Providing practical support related to jobs				
Network size	0.94 (0.74-1.19)	0.95 (0.74-1.21)	0.94 (0.74-1.20)	0.92 (0.71-1.19)
Percentage of network members who are daily contact	1.00 (0.97-1.03)	1.00 (0.97-1.03)	1.00 (0.97-1.03)	1.00 (0.97-1.03)
Percentage of network members who are family members	1.02 (0.99-1.04)	1.01 (0.99-1.04)	1.01 (0.99-1.04)	1.01 (0.99-1.04)
Percentage of network members who are friends	1.01 (0.93-1.10)	1.01 (0.93-1.11)	1.01 (0.93-1.10)	1.01 (0.92-1.11)
Percentage of network members who are living in the same household	0.99 (0.97-1.01)	0.99 (0.97-1.01)	1.00 (0.98-1.02)	1.00 (0.98-1.02)
Percentage of network members who are having hypertension	0.98^∗∗^ (0.96-1.00)	0.98^∗∗^ (0.96-1.00)	0.98^∗∗^ (0.96-1.00)	0.98^∗∗^ (0.96-1.00)
Living with spouse/partners (yes vs no)	1.03 (0.41-2.60)	1.03 (0.41-2.62)	1.25 (0.47-3.32)	1.42 (0.52-3.88)
Participation in social activities (yes vs no)	0.77 (0.38-1.58)	0.79 (0.38-1.63)	0.79 (0.37-1.68)	0.78 (0.35-1.75)

^∗^ *p* < 0.1; ^∗∗^*p* < 0.05; ^∗∗∗^*p* < 0.01; ^a^reference group was controlled hypertension; ^b^model 1 was adjusted for age and sex; ^c^model 2 was adjusted for age, sex, and quality of life; ^d^model 3 was adjusted for age, sex, quality of life, body mass index, and number of comorbidities; ^e^model 4 was adjusted for age, sex, quality of life, body mass index, number of comorbidities, smoking, and alcohol use.

**Table 3:** Relationship between social network characteristics and behavioral adherence.

Characteristics of social network	Behavioral adherence score			
	Coef. (95% CI)	Coef. (95% CI)	Coef. (95% CI)	Coef. (95% CI)
	Model 1^a^	Model 2^b^	Model 3^c^	Model 4^d^
Total network size	0.20 (-0.16-0.57)	0.16 (-0.21-0.53)	0.15 (-0.23-0.52)	0.14 (-0.23-0.50)
Providing informational support related to advice				
Network size	-0.10 (-0.41-0.21)	-0.09 (-0.40-0.22)	-0.09 (-0.40-0.22)	-0.10 (-0.40-0.20)
Percentage of network members who are daily contact	-0.01 (-0.04-0.03)	-0.00 (-0.04-0.03)	0.00 (-0.03-0.04)	0.00 (-0.03-0.04)
Percentage of network members who are family members	0.03 (-0.01-0.06)	0.03^∗^ (-0.01-0.07)	0.03 (-0.01-0.06)	0.02 (-0.01-0.06)
Percentage of network members who are friends	0.01 (-0.03-0.04)	0.01 (-0.02-0.05)	0.01 (-0.03-0.04)	0.01 (-0.03-0.04)
Percentage of network members who are living in the same household	-0.06^∗∗∗^ (-0.10-0.02)	-0.07^∗∗∗^ (-0.12-0.03)	-0.07^∗∗∗^ (-0.11-0.03)	-0.08^∗∗∗^ (-0.12-0.03)
Percentage of network members who are having hypertension	0.01 (-0.03-0.05)	0.01 (-0.04-0.05)	0.01 (-0.04-0.05)	0.01 (-0.03-0.05)
Providing emotional support related to discomfort				
Network size	-0.00 (-0.31-0.30)	0.00 (-0.30-0.31)	0.01 (-0.30-0.31)	0.04 (-0.26-0.33)
Percentage of network members who are daily contact	-0.01 (-0.04-0.03)	0.01 (-0.03-0.04)	0.01 (-0.03-0.04)	-0.00 (-0.04-0.03)
Percentage of network members who are family members	0.02 (-0.01-0.06)	0.00 (-0.04-0.05)	0.01 (-0.04-0.05)	0.01 (-0.03-0.05)
Percentage of network members who are friends	-0.01 (-0.04-0.02)	-0.02 (-0.06-0.01)	-0.02 (-0.06-0.01)	-0.02 (-0.05-0.01)
Percentage of network members who are living in the same household	-0.01 (-0.04-0.02)	-0.01 (-0.04-0.02)	-0.01 (-0.05-0.02)	-0.01 (-0.05-0.02)
Percentage of network members who are having hypertension	0.03^∗^ (-0.00-0.06)	0.03^∗^ (-0.00-0.06)	0.03^∗^ (-0.00-0.06)	0.03^∗^ (-0.00-0.06)
Providing emotional support related to decisions				
Network size	-0.30^∗^ (-0.60-0.01)	-0.31^∗∗^ (-0.62-0.01)	-0.32^∗∗^ (-0.63-0.01)	-0.26^∗^ (-0.56-0.04)
Percentage of network members who are daily contact	0.04^∗∗^ (0.00-0.08)	0.03 (-0.01-0.07)	0.03 (-0.01-0.07)	0.03^∗^ (-0.01-0.07)
Percentage of network members who are family members	-0.02 (-0.05-0.02)	-0.01 (-0.05-0.03)	-0.01 (-0.05-0.03)	-0.01 (-0.05-0.02)
Percentage of network members who are friends	-0.04 (-0.13-0.05)	-0.03 (-0.12-0.06)	-0.03 (-0.12-0.06)	-0.04 (-0.12-0.04)
Percentage of network members who are living in the same household	-0.02 (-0.05-0.01)	-0.02 (-0.05-0.01)	-0.02 (-0.05-0.01)	-0.02 (-0.05-0.01)
Percentage of network members who are having hypertension	0.01 (-0.03-0.04)	0.01 (-0.03-0.04)	0.01 (-0.03-0.04)	0.01 (-0.03-0.04)
Providing practical support related to sickness				
Network size	-0.45∗^∗^ (-0.86-0.04)	-0.42^∗∗^ (-0.83-0.01)	-0.39^∗^ (-0.80-0.02)	-0.34^∗^ (-0.74-0.06)
Percentage of network members who are daily contact	-0.03 (-0.07-0.01)	-0.03 (-0.07-0.01)	-0.04^∗^ (-0.08-0.01)	-0.03 (-0.07-0.01)
Percentage of network members who are family members	0.04 (-0.01-0.09)	0.04 (-0.01-0.09)	0.04 (-0.01-0.09)	0.03 (-0.02-0.08)
Percentage of network members who are friends	0.12^∗∗∗^ (0.05-0.19)	0.12^∗∗∗^ (0.05-0.19)	0.12^∗∗∗^ (0.05-0.18)	0.10^∗∗∗^ (0.04-0.17)
Percentage of network members who are living in the same household	0.00 (-0.03-0.04)	0.00 (-0.03-0.04)	0.01 (-0.02-0.04)	0.01 (-0.02-0.04)
Percentage of network members who are having hypertension	-0.02 (-0.06-0.03)	-0.02 (-0.06-0.03)	-0.02 (-0.06-0.03)	-0.03 (-0.07-0.02)
Providing practical support related to jobs				
Network size	0.05 (-0.40-0.50)	0.05 (-0.41-0.50)	0.04 (-0.42-0.49)	0.03 (-0.41-0.46)
Percentage of network members who are daily contact	-0.01 (-0.07-0.05)	-0.01 (-0.07-0.06)	-0.01 (-0.07-0.05)	-0.01 (-0.07-0.05)
Percentage of network members who are family members	0.02 (-0.03-0.07)	0.03 (-0.02-0.08)	0.03 (-0.02-0.08)	0.03 (-0.02-0.08)
Percentage of network members who are friends	0.18^∗∗∗^ (0.04-0.31)	0.18^∗∗∗^ (0.05-0.32)	0.19^∗∗∗^ (0.06-0.32)	0.19^∗∗∗^ (0.06-0.32)
Percentage of network members who are living in the same household	-0.01 (-0.05-0.03)	-0.02 (-0.06-0.03)	-0.02 (-0.06-0.03)	-0.02 (-0.06-0.02)
Percentage of network members who are having hypertension	-0.02 (-0.05-0.02)	-0.02 (-0.05-0.02)	-0.02 (-0.05-0.02)	-0.01 (-0.05-0.02)
Living with spouse/partners (yes vs no)	1.20 (-0.87-3.26)	1.30 (-0.76-3.36)	1.18 (-0.90-3.25)	0.77 (-1.23-2.78)
Participation in social activities (yes vs no)	2.54^∗∗∗^ (0.96-4.11)	2.22^∗∗∗^ (0.63-3.81)	2.29^∗∗∗^ (0.70-3.89)	2.56^∗∗∗^ (0.99-4.13)

^∗^ *p* < 0.1; ^∗∗^*p* < 0.05; ^∗∗∗^*p* < 0.01; ^a^model 1 was adjusted for age and sex; ^b^model 2 was adjusted for age, sex, and quality of life; ^c^model 3 was adjusted for age, sex, quality of life, body mass index, and number of comorbidities; ^d^model 4 was adjusted for age, sex, quality of life, body mass index, number of comorbidities, smoking, and alcohol use.

## Data Availability

A data availability statement is compulsory for research articles and clinical trials. Here, requests for access to individual subject data may be made to Nguyen Hoang Thanh: please send an email to nguyenht.hmu@gmail.com| nguyenhoangthanh@hmu.edu.vn.
